# The Eyes Have It—Or Do They?

**DOI:** 10.4269/ajtmh.17-0045

**Published:** 2017-05-03

**Authors:** Susan Lewallen, Terrie Taylor

**Affiliations:** 1 Kilimanjaro Centre for Community Ophthalmology, University Cape Town Department of Ophthalmology, Groote Schuur Hospital, Observatory, Cape Town, South Africa; 2 Department of Osteopathic Medical Specialties, College of Osteopathic Medicine, Michigan State University, East Lansing, Michigan; 3 Blantyre Malaria Project, University of Malawi College of Medicine, Blantyre, Malawi

**Figure 1.**
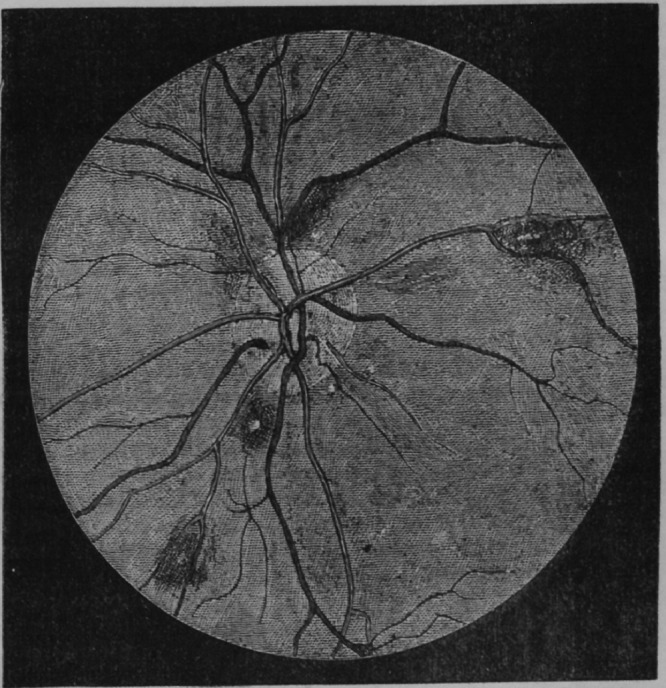
An early image of malarial retinopathy.

The reference to “classic” malarial retinopathy in the article by Villaverde and others^1^ marks a watershed for some of us; it was not always so. Malarial retinopathy might have gone unnoticed for many more years than it did. Its recognition was not the result of a large well-funded grant but rather a serendipitous conversation between friends, over dinner in Blantyre, Malawi, in 1990. Observations by nonophthalmologist clinicians through undilated pupils with direct ophthalmoscopes had revealed hemorrhages, originally described in 1877 by Mackenzie^2^ (Figure 1

). In 1990, these older observations were superseded, postprandially, by assessments carried out by an ophthalmologist armed with an indirect ophthalmoscope, through pupils dilated with short-acting pharmacologic agents. She quickly corroborated the presence of hemorrhages, but also noted two additional findings: abnormally colored retinal vessels in a seemingly random distribution, unlike those seen in other retinal vascular conditions, and patchy retinal whitening, similar in nature to the opacification in common retinal vein occlusions but in a markedly different distribution.

The next steps were slow: the ophthalmologist was the only ophthalmologist in the southern region of Malawi, so direct corroboration was difficult. Retinal photography was not a possibility there at the time. The first images, captured on a borrowed fundus camera in children with roving eye movements, developed in the United Kingdom and posted back to Malawi, were too indistinct to be convincing. Systematic documentation of the findings in a consecutive series of children, with analysis of their association with outcome was more convincing, however, and led to the first publication describing the range of findings and linking them to outcome in 1993.^3^ Further systematic observations via a rotating schedule of volunteer ophthalmologists in the context of an autopsy-based study coupled with better cameras and imaging techniques (available through a collaboration with Simon Harding, University of Liverpool), revealed the histopathological correlates of malarial retinopathy.^4^–^6^

In the autopsy study,^7^ malarial retinopathy was associated with the sequestration of parasitized red cells in the microvasculature of children who met the standard World Health Organization (WHO) definition of cerebral malaria (CM)—this same definition is used by Villaverde and others.^1^ No extracerebral causes of death were identified by postmortem examination in these patients. By contrast, in patients who met the standard WHO definition of CM but had no evidence of malarial retinopathy, a nonmalarial cause of death was always identified, and there was little or no sequestration of parasitized red cells in the postcapillary venules of the brain.

In a comatose, parasitemic patient, the presence of malarial retinopathy greatly improves the specificity of the clinical diagnosis of CM, and has been useful for studies of malaria pathogenesis. Given that sequestration of erythrocytes with mature parasites is a feature of all *Plasmodium falciparum* infections, it is not surprising that malarial retinopathy has been observed in other clinical malaria syndromes.^8^ This does not detract from its utility in patients with clinically defined CM.

Interpreting the absence of malarial retinopathy in these same patients is the focus of Villaverde and others.^1^ The Villaverde study assesses clinical and laboratory variables in children with CM, subdivided into retinopathy-positive or retinopathy-negative groups. Retinopathy-positive children generally had more severe illness and a larger percentage of them died, although this was not statistically significant in the study. The Malawi autopsy study suggested that the malaria infections in retinopathy-negative patients who died were incidental, and unrelated to the ultimate cause of death. The sample size in the autopsy study is small, and, critically, it is derived from the extreme of the disease severity spectrum. Much remains to be learned about the parasitological and immunological basis of asymptomatic parasitemia and at present, it is not possible to determine the cause-and-effect relationship between a malaria infection and concomitant symptoms at the level of the individual patient.

One possible explanation for retinopathy-negative children in any setting, supported by the Villaverde data as well as by a large study on clinical severity,^9^ is that the parasitemia is causally related to the clinical symptoms, and that children with retinopathy-negative CM are simply at an earlier, less severe, preretinopathy stage of their disease. If left untreated and observed over time (which is clearly unethical), this group would ultimately develop malarial retinopathy.

Another strong possibility, indeed a probability, is that our current technique for identifying malarial retinopathy, binocular indirect ophthalmoscopy, lacks sufficient sensitivity. It also relies heavily on the experience of the observer and requires subjective judgments: Villaverde and others^1^ note a 15.6% discordance rate between the classifications when more than one examiner was involved. Algorithmically driven analyses of ocular fundus images have improved the diagnosis of diabetic retinopathy; would a similar approach obviate the need for clinical judgment in assessing the ocular fundi of children with CM? Just as the indirect ophthalmoscope was a dramatic improvement over the direct ophthalmoscope, and polymerase chain reaction–based techniques for detecting malaria parasites expanded the ranks of those classified as “infected,” newer, more sensitive imaging techniques will likely identify patients whose malarial retinopathy is too subtle to be identified using the standard approach.^10^

Conditions that exist on a continuous spectrum, such as malarial retinopathy and the clinical manifestations of a malaria illness, are difficult to categorize. The pathophysiologic processes responsible for the clinical signs must reach a threshold before they become manifest. Some of the early consternation experienced back in 1990 in trying to describe and categorize malarial retinopathy was due to the wide clinical spectrum in which it occurs. It is good to be reminded by Villaverde and others^1^ that clinically diagnosed CM itself exists on a spectrum within severe malaria syndromes.
